# Pentachlorophenol has significant adverse effects on hematopoietic and immune system development in zebrafish (*Danio rerio*)

**DOI:** 10.1371/journal.pone.0265618

**Published:** 2022-03-25

**Authors:** Aleeza Namit, William Dowell, Sandrine Matiasek, Jackson Webster, David L. Stachura

**Affiliations:** 1 Department of Biological Sciences, California State University Chico, Chico, CA, United States of America; 2 Department of Geological and Environmental Sciences, California State University Chico, Chico, CA, United States of America; 3 Department of Civil Engineering, California State University Chico, Chico, CA, United States of America; University of Mississippi Medical Center, UNITED STATES

## Abstract

In November 2018, the Camp Fire devastated the mountain community of Paradise, CA. The burning of plastic pipes, wiring, construction materials, paint, and car batteries released toxic chemicals into the environment, contaminating the air, soil, and local waterways. Examples of toxins that were identified in the creeks and waterways in and around Paradise included pentachlorophenol (PCP), chrysene, and polyaromatic hydrocarbons. The effects of some of these chemicals on embryonic development, hematopoiesis (blood formation), and the immune system have not been thoroughly studied. Defining safe levels and the long-term effects of exposure is imperative to understanding and mitigating potential negative future outcomes. To perform these studies, we utilized zebrafish (*Danio rerio*), a commonly used vertebrate model system to study development. We observed the adverse effects of PCP on the development of zebrafish by using fluorescence microscopy, and saw that increased concentrations of PCP decreased the numbers of normal red blood cells and myeloid cells. Additionally, we observed that animal survival decreased in response to increasing concentrations of PCP. Furthermore, the prevalence of characteristic physical deformities such as tail curvature were greater in the treatment groups. Lastly, *runx1*, *cmyb*, and *cd41* expression was reduced in fish treated with PCP. These results suggest that PCP has a previously underappreciated effect on blood and immune cell development and future studies should be performed to determine the molecular mechanisms involved.

## Introduction

Hematopoiesis is the process by which all mature blood and immune cells are generated from pluripotent hematopoietic stem and progenitor cells (HSPCs). This highly regulated process is well conserved across vertebrate species; teleosts (bony fish) and humans share the same molecular pathways responsible for blood formation and homeostasis [reviewed in 1–3]. *Danio rerio* (zebrafish) are an excellent teleost model organism for studying hematopoiesis because hematopoietic-specific fluorescent transgenic fish lines exist. Additionally, zebrafish are fecund, allowing large sample sizes. Lastly, zebrafish embryos develop externally, allowing observations to be made during the early developmental stages of the animal. For all of these reasons, zebrafish are an excellent model to analyze the effects of drugs and toxins on zebrafish hematopoiesis [reviewed in 4]. Due to the similarity of molecular pathways between humans and zebrafish, we can also extrapolate these findings to early human hematopoietic development and perturbation.

The burning of plastics, paints, treated wood, and other materials can release debris containing volatile organic compounds into the environment [5–9]. Following the Camp Fire, which destroyed the town of Paradise, CA in 2018, a panel of chemical contaminants were identified in creeks of Paradise during subsequent storms. Among the chemicals identified was pentachlorophenol (PCP; Zhao et al., in preparation). PCP is a commonly used wood preservative, especially in telephone poles and railroad ties, in much of California and the United States [10]. When wood treated with PCP was burned in the Camp Fire, it is likely that toxic ash was created and transported into the surrounding environment. A proportion of ash released during a wildfire can migrate into waterways that surround the area [reviewed in 11], suggesting that ash containing PCP may have landed in waterways surrounding Paradise and contaminated the water. Overall, anthropogenic climate change has resulted in a warmer, drier climate in much of California [12]. These conditions have resulted in an increasing incidence of preconditions associated with wildfires [13]. Given the encroachment of human settlement on wilderness, termed the Wildland Urban Interface (WUI) [14], a future severe PCP contamination event resulting from a destructive wildfire is a distinct possibility. In order to mitigate unforeseen consequences of wildfires, we wanted to analyze the effects that PCP contamination had on vertebrate development.

Epidemiological studies have sought to establish a relationship between PCP exposure and hematopoietic cancers such as non-Hodgkin’s lymphoma and myeloma. A meta-analysis conducted in 2008 suggested that PCP exposure was associated with an increased risk of hematopoietic cancer [15]. Furthermore, PCP exposure has been implicated in inhibiting the ability of natural killer (NK) cells to secrete TNFα [16] and with lower levels of CD4^+^ and CD8^+^ T cells [17]. However, the effects that PCP has on early hematopoietic development, particularly on cells of the erythroid and myeloid lineage, are enigmatic. While there is no direct link between cancer and embryonic hematopoiesis per se, many of the same molecular pathways are involved, and their dysregulation is involved in both development and disease. Consequently, we sought to identify the effects PCP has on the erythroid and myeloid lineage of blood cells and developing zebrafish embryos, in order to discover its possible impacts on wildlife and human health.

## Materials and methods

### Zebrafish husbandry and care

Zebrafish were mated, staged, and raised as described [18] and maintained in accordance with California State University, Chico (CSUC) Institutional Animal Care and Use Committee (IACUC) guidelines. All procedures were approved by the CSUC IACUC before being performed. Personnel were trained in animal care by taking the online Citi Program training course entitled “Working With Zebrafish (*Danio rerio*) in Research Settings” (https://www.citiprogram.org). *mpx*:EGFP [19] and *gata1*:DsRed [20] fish were used for these studies. Zebrafish were housed in a 700 L recirculating zebrafish aquarium system (Aquatic Enterprises, Seattle, WA) regulated by a Profilux 3 Outdoor module that regulated salinity, pH, and temperature (GHL International, Kaiserslautern, Germany) 24-hours-a day. The facility was illuminated on a 14-hour light/ 10-hour dark cycle. Zebrafish were fed once a day with hatched brine shrimp (Brine Shrimp Direct, Ogden, UT) and once a day with Size 4 Zebrafish Research Diet (daniolab, Boston, MA).

### Microscopy

All observations were made with a Leica M165C microscope and pictures were taken with a Leica DFC295 camera. *gata1*:DsRed, *mpx*:GFP double positive embryos were treated with PCP concentrations of 0, 25, 50, 75, and 100 μg/L at time points of 0, 24, 48, and 72 hours post fertilization (hpf). Embryos were observed and imaged 24 hours after their treatment was applied. In other words: embryos treated at 0 hpf were examined at 24 hpf, embryos treated at 24 hpf were examined at 48 hpf, embryos treated at 48 hpf were examined at 72 hpf, and animals treated at 72 hpf were examined at 96 hpf. *gata1*:DsRed embryos were visualized and counted; they were grouped together as having normal red blood cell numbers, less red blood cells, or no red blood cells. Images of *mpx*:GFP embryos were taken and the number of fluorescent cells per embryo were enumerated. To perform this, images were taken at the same exposure and every *mpx*:GFP^+^ cell was manually counted. To prevent bias, the images were coded and students counted the *mpx*:GFP^+^ blinded from the experimental variable.

### 2,3,4,5,6-pentachlorophenol (PCP)

PCP was dissolved in methanol at a concentration of 500 μg/mL. Fish embryos were treated with PCP concentrations of 0, 25, 50, 75, and 100 μg/L at time points of 0, 24, 48, and 72 hpf. Animals were kept in a dark incubator. Vehicle control trials were performed to ensure methanol was not the toxic agent responsible for data that were collected.

### Quantitative reverse transcriptase PCR (qRT-PCR)

RNA was extracted with the RNeasy Plus Mini Kit (Qiagen, Hilden, Germany) from untreated embryos and those treated with PCP at 24 and 48 hours. Genomic DNA was removed from samples with gDNA eliminator mini spin columns (Qiagen, Hilden, Germany). iScript (Bio-Rad, Hercules, CA) was used to generate cDNA from each sample. Each cDNA sample was made by pooling ten random whole embryos per condition. SsoAdvanced Universal SYBR green mastermix (Bio-Rad, Hercules, CA) was used to amplify genes, with an Eppendorf realplex2 (Eppendorf, Hamburg, Germany) machine using primers for *ef1α* [21], *runx1* [21], *cmyb* [21], and *itga2b* (commonly referred to as *cd41)* [21]. All PCR primers were validated previously and designed to span exons. Data were analyzed for relative expression change with *ef1α* as the reference gene. ΔΔCt was calculated by comparing the expression of the treated embryos to control embryos and to the reference gene, *ef1α*.

### Statistical methods

Statistical analyses were performed in Microsoft Excel. To discern statistical difference, data were analyzed using an unpaired two-tailed Student’s T test assuming unequal variance. All raw data from these studies are supplied in **S1 File**.

## Results

**Fig 1:**
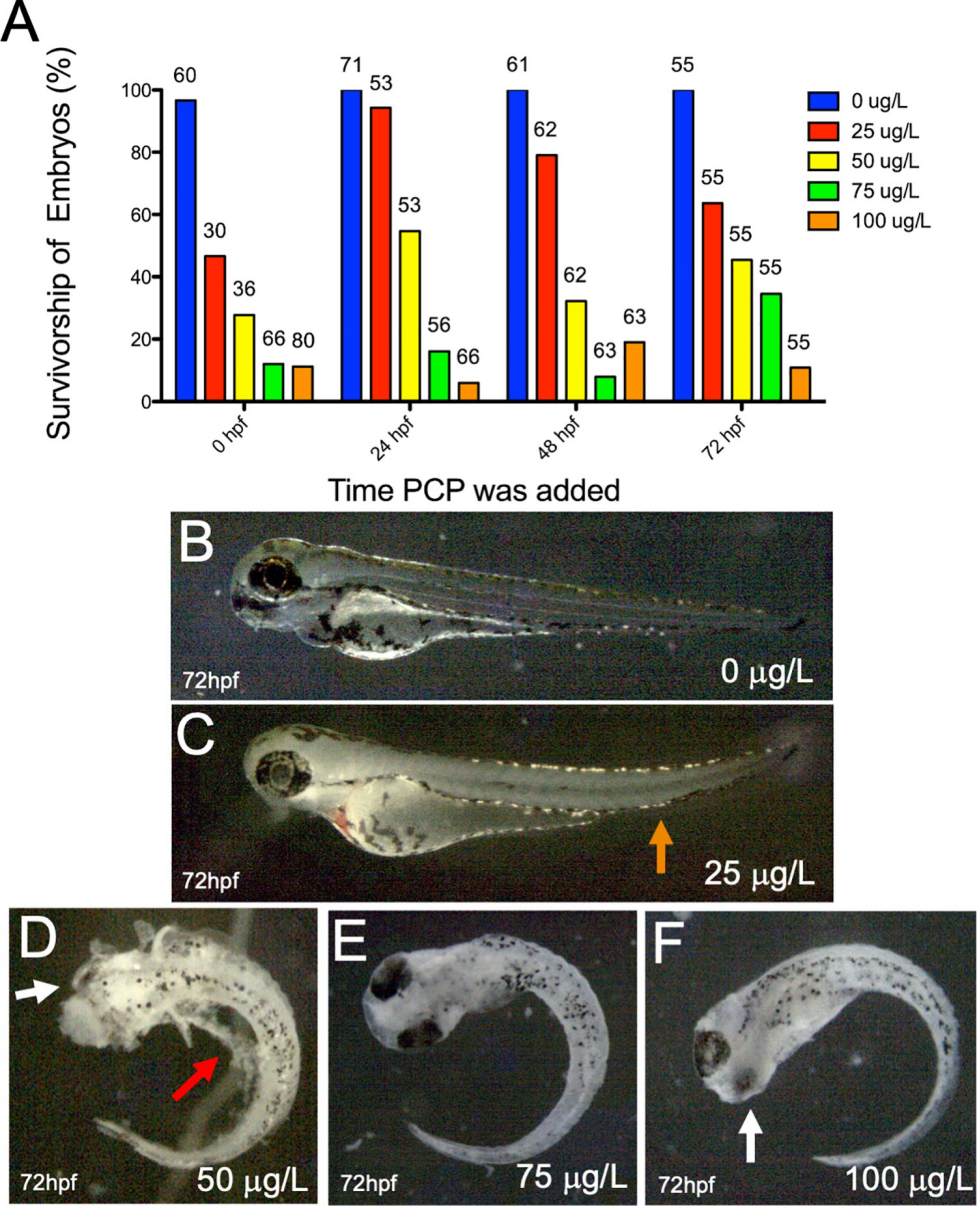
Survivorship of zebrafish embryos decreased and malformations increased across all treatment time periods as PCP concentrations increased. A) PCP was added to zebrafish embryos at 4 distinct time points: 0, 24, 48, and 72 hpf. PCP was administered at concentrations of 0 (blue), 25 (red), 50 (yellow), 75 (green), and 100 (orange) μg/L. All embryos were analyzed 24 hours after PCP administration. Numbers above bars represent how many embryos were analyzed for that condition. B) Example of a 72 hpf zebrafish treated with 0 μg/L PCP at 48 hpf. C) Example of a 72 hpf zebrafish treated with 25 μg/L PCP at 48 hpf. Orange arrow denotes abnormal tail curvature. D) Example of a 72 hpf zebrafish treated with 50 μg/L PCP at 48 hpf, with eye malformation (white arrow) and necrotic tissue (red arrow). E) Example of a 72 hpf zebrafish treated with 75 μg/L PCP at 48 hpf that had body axis curvature. F) Example of a 72 hpf zebrafish treated with 100 μg/L PCP at 48 hpf that had body axis curvature and eye malformations (white arrow).

To examine and establish a relationship between increasing concentrations of PCP and its effects on zebrafish survival, we added 0, 10, 25, 50, 75, and 100 μg/L of PCP at four distinct time points during development including 0, 24, 48, and 72 hpf. All embryos were examined 24 hours after PCP addition, and the survival of the embryos was observed under an inverted microscope. To qualify as “alive,” zebrafish embryos had to have a visible heartbeat and circulation. They also had to be either moving their pectoral fins or respond to stimuli such as tapping or light exposure. As expected, untreated embryos showed the highest survival rate in all categories. 10 μg/L had no effect on survival, so we added increasing amounts of PCP to the embryos. As increasing concentrations of PCP were added, there was a clear downward trend in survivorship, regardless of what time PCP was added to the embryos (**Fig 1A**). Lowest rates of survivorship were observed in the 0 hpf category, when PCP was added immediately after embryo fertilization. At all four timepoints, embryos treated with 50, 75, and 100 μg/L PCP showed a considerably lower survival rate compared to embryos in the 0 and 25 μg/L treatments (**Fig 1**). Due to the fact that PCP is dissolved in methanol, we also added methanol to embryos at corresponding dilutions used with PCP. No differences were seen when compared to untreated embryos, indicating that the effects were due to PCP and not the vehicle. These findings indicate that PCP has clear negative effects on zebrafish survival, regardless of when it was added to the embryo.

While we observed a decrease in survivorship as PCP concentrations increased, we also observed other physical deformities by 72 hpf. While untreated animals had straight tails (**Fig 1B),** tail curvature was obvious when PCP was added to embryos (**Fig 1C,** orange arrow), and embryos were more affected as PCP concentrations increased (**Fig 1D–1F)**. Many embryos also had extensive necrotic tissue and malformations (**Fig 1D–1F,** red and white arrows). In these examples, animals represented in **Fig 1B** and **1C** were categorized as “alive” (even though **Fig 1C** showed tail curvature), while embryos shown in **Fig 1D–1F** were not. In essence, increased levels of PCP appear to affect not only survival, but also embryonic morphology.

**Fig 2:**
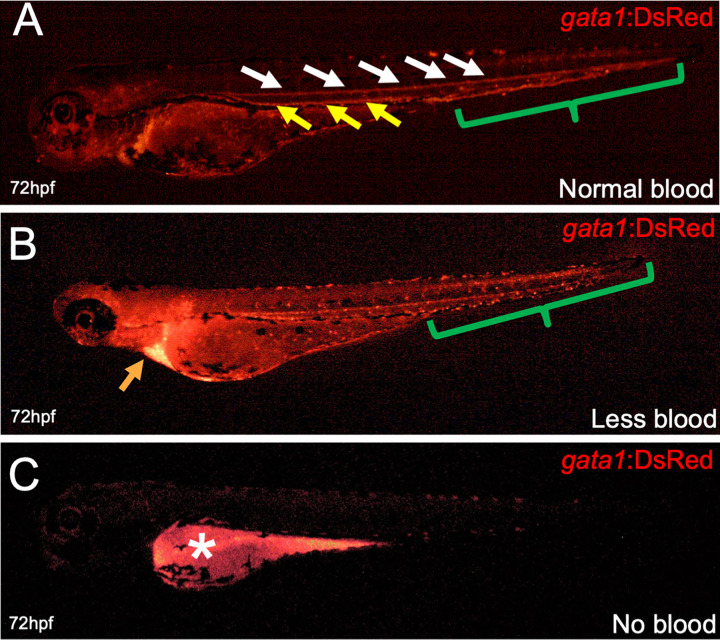
Representative images of red blood cells after PCP addition. *gata1*:DsRed embryos were treated with 25 μg/L PCP at 48 hpf and fluorescence was analyzed 24 hours later at 72 hpf. (A) “Normal” blood development was characterized by continuous circulation and fluorescence in the dorsal aorta (white arrows), cardinal vein (yellow arrows), and the caudal hematopoietic tissue (green bracket). (B) “Less blood” was characterized by a non-continuous stream of fluorescent cells and visibly reduced blood circulation and pooling of blood in the Ducts of Cuvier (orange arrow). (C) “No blood” was characterized by the absence of fluorescent blood cells in the fish and no observable circulation. White star denotes autofluorescence in the yolk ball.

**Fig 3:**
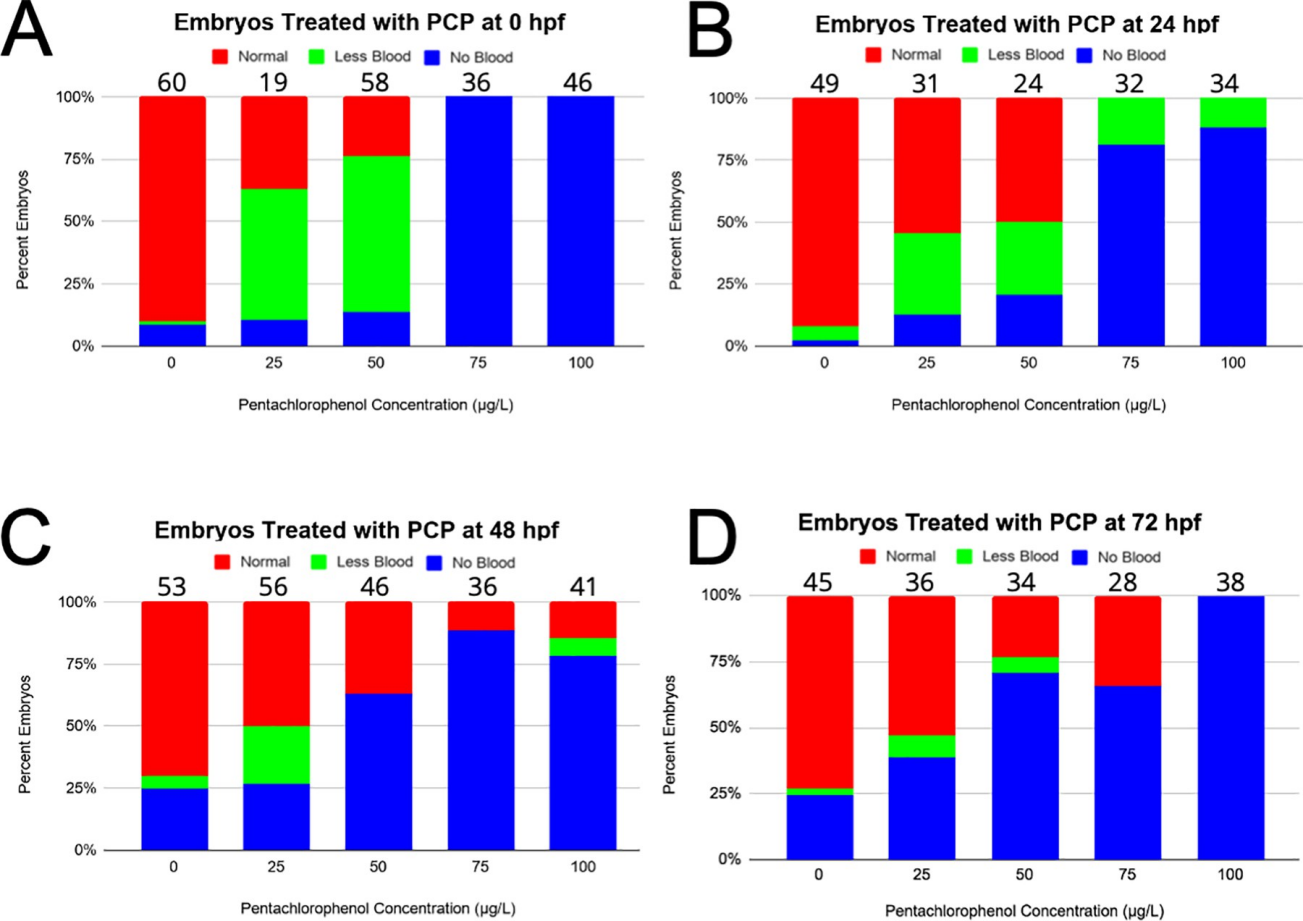
Blood development and circulation is negatively affected by the addition of PCP in the 0, 24, 48, and 72 hpf treatment groups. Blood analysis of zebrafish embryos treated with PCP was analyzed 24 hours after drug application. *gata1*:DsRed transgenic zebrafish and fluorescent microscopy were used to observe blood circulation. Blood development in embryos treated at 0 (A), 24 (B), 48 (C), and 72 (D) hpf. Normal circulation (red), less blood (green), and no blood (blue) are all phenotypes highlighted in **Fig 2**. Numbers of embryos observed are listed above all columns.

As PCP did not kill all of the embryos exposed, we wanted to observe if the surviving animals had issues with hematopoietic development. To do this, we only examined fish that were alive and morphologically normal (see above). First, we observed red blood cells (erythrocytes), which are essential for transport of oxygen to distant tissues and are the first blood cells to be produced in the developing embryo. To characterize the effect PCP had on the development of erythrocytes, we utilized *gata1*:DsRed transgenic zebrafish [20], which have the erythroid-specific *gata1* promoter driving a red fluorescent protein, making red blood cells fluoresce. Administering PCP to the embryos at various concentrations and time points allowed us to analyze blood development in real time under a fluorescent microscope. We observed that most untreated embryos had normal blood formation and circulation (**Fig 2A**); when visualized under a microscope the dorsal aorta, caudal hematopoietic tissue, cardinal vein, and heart were full of fluorescent blood cells. When imaged, this appears as a solid red line of blood cells (**Fig 2A**). However, many fish treated with PCP exhibited less blood, characterized by a non-continuous stream of fluorescent cells and visibly reduced numbers of blood cells, sometimes accompanied by blood pooling in the Ducts of Cuvier before entering the heart (**Fig 2B**). Other embryos were completely devoid of fluorescent blood cells, even though they clearly had a heartbeat and cells present in their vasculature (**Fig 2C**). Quantitation of these phenotypes indicated that as increasing amounts of PCP were added to 0, 24, 48, and 72 hpf embryos, the amounts of live fish with abnormal blood counts increased dramatically (**Fig 3**). Importantly, at concentrations of 75 and 100 μg/L, mostly all live fish had no red blood cells visible at all. Importantly, fish with “less blood” never recovered normal levels of blood, indicating that the PCP did not just cause a delay in red blood cell generation. Overall, these findings indicate that as PCP concentrations increased, regardless of when it was administered, red blood cell development was negatively impacted.

**Fig 4:**
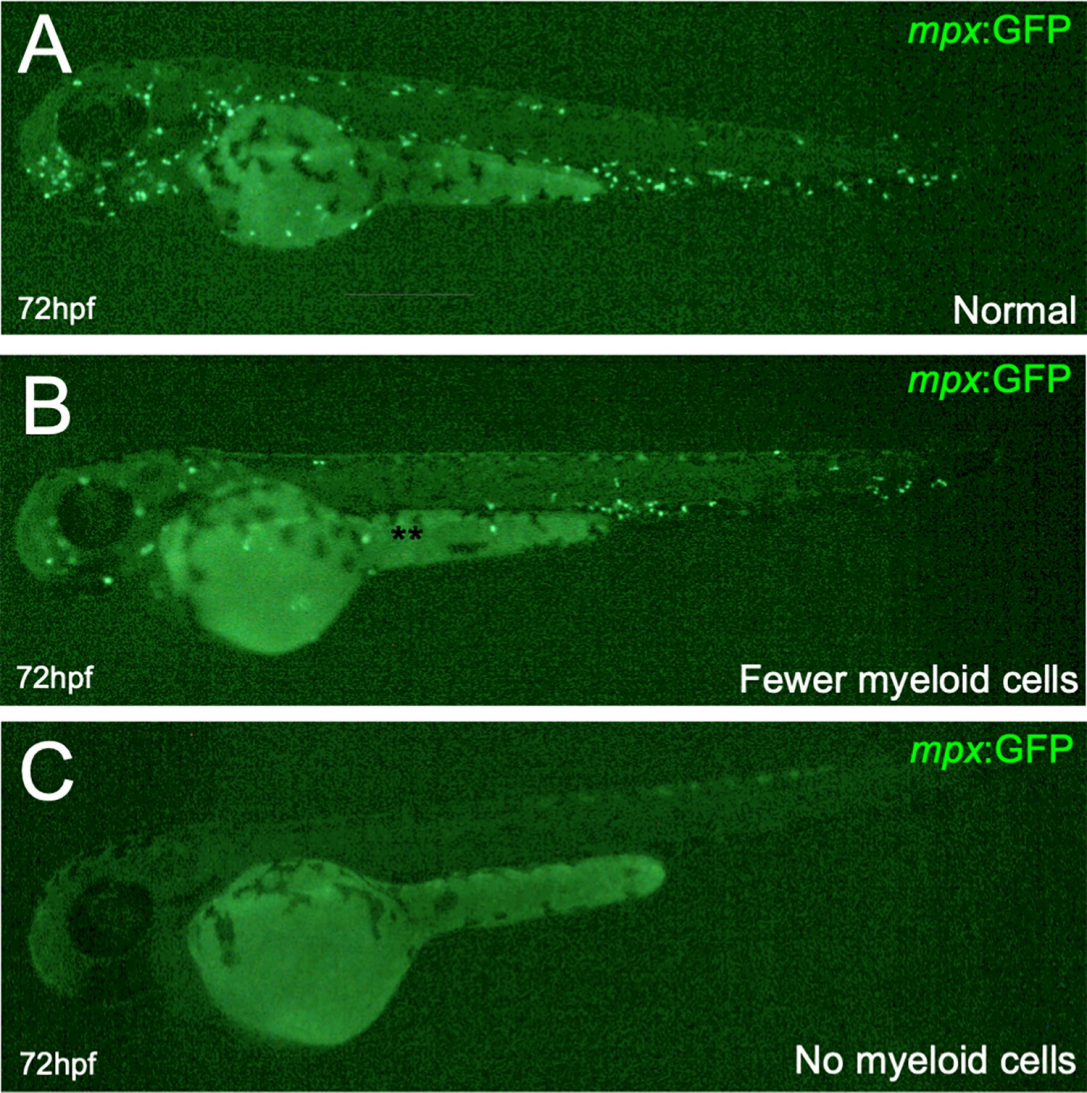
Representative images of myeloid cells after PCP addition. *mpx*:GFP embryos were treated with 50 μg/L PCP at 48 hpf and fluorescence was analyzed 24 hours later at 72 hpf. Representative images showing normal (A), reduced (B), and no myeloid cells (C).

**Fig 5:**
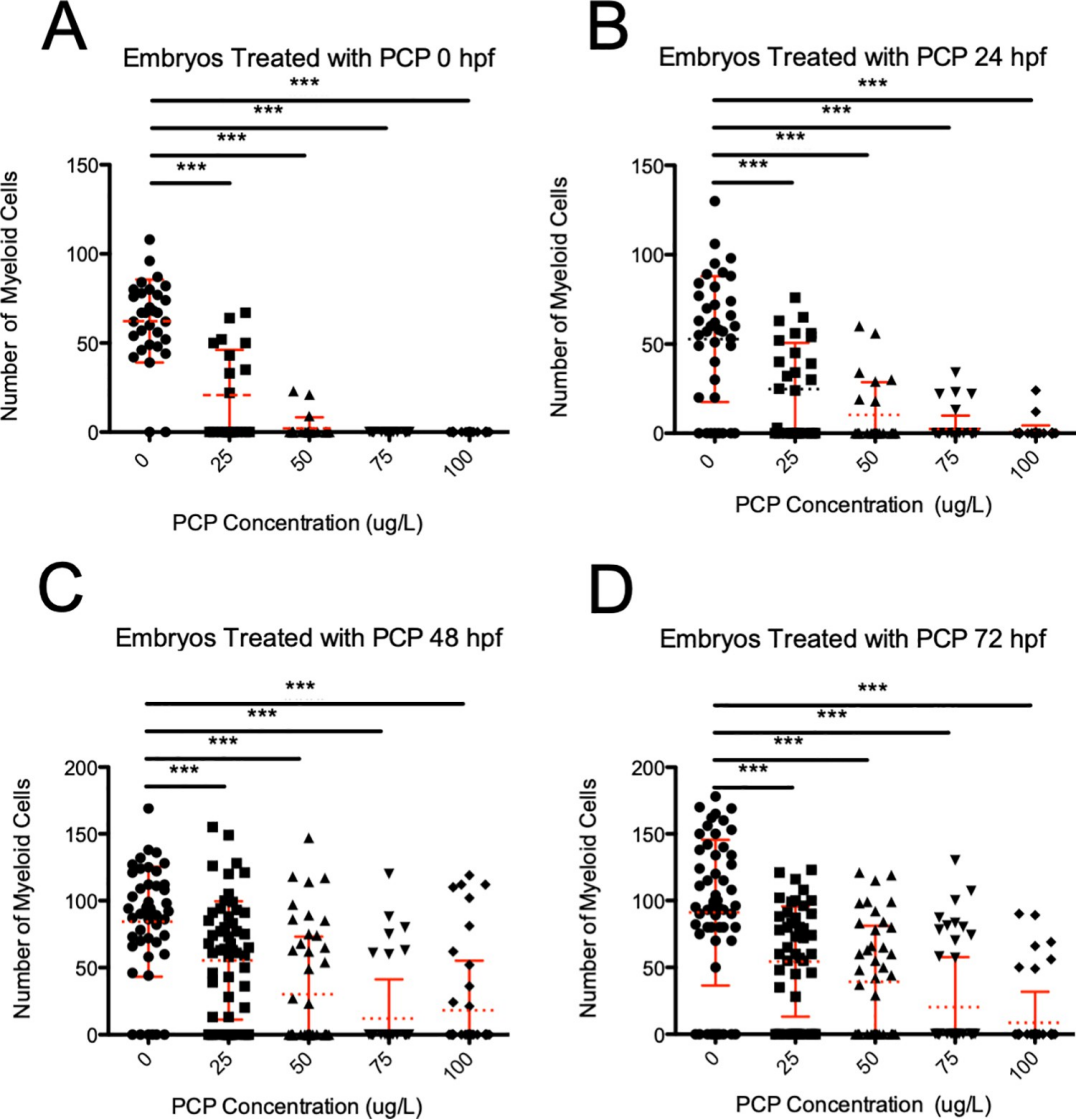
Myeloid cell development is negatively affected by the addition of PCP in the 0, 24, 48, and 72 hpf treatment groups. Blood analysis of live zebrafish embryos treated with PCP was analyzed 24 hours after drug application. *mpx*:*GFP* transgenic zebrafish and fluorescent microscopy were used to observe neutrophils (see **Fig 4**). Blood development in embryos was quantitated after PCP treatment at 0 (A), 24 (B), 48 (C), and 72 hpf (D). Every point denotes the numbers of myeloid cells present in one animal. Dashed line represents average, and error bars represent SD. *** denotes p < 0.001.

To determine if PCP also had a negative effect on the formation of myeloid cells, we utilized *mpx*:GFP transgenic zebrafish [19] that have the myeloid-specific promoter *mpx* driving expression of a green fluorescent protein. *mpx*:GFP mainly marks neutrophils, which are the most abundant leukocytes in the animal that protects it from bacterial infection. Again, we utilized only live fish for these analyses; fish that were obviously necrotic, severely morphologically defective, lacking a heartbeat or normal physiological response to stimuli were excluded from these studies. To live fish, we administered PCP to embryos at 0, 24, 48, and 72 hpf. Normal numbers of myeloid cells were observed when no PCP was added (**Fig 4A**). However, we observed several deleterious phenotypes when PCP was added, including fewer myeloid cells (**Fig 4B**) and animals with no myeloid cells at all (**Fig 4C**). Because these myeloid cells are tissue-resident, we then counted the numbers of myeloid cells present. Embryos treated at 0 hpf were drastically affected, with animals treated with 75 or 100 μg/L having no neutrophils (**Fig 5A**). Animals had more neutrophils when PCP was added at later time points, but all had a significant reduction of myeloid cells (**Fig 5B–5D**). These data indicate that PCP exposure had a severe negative effect on neutrophil numbers in the developing embryo.

**Fig 6:**
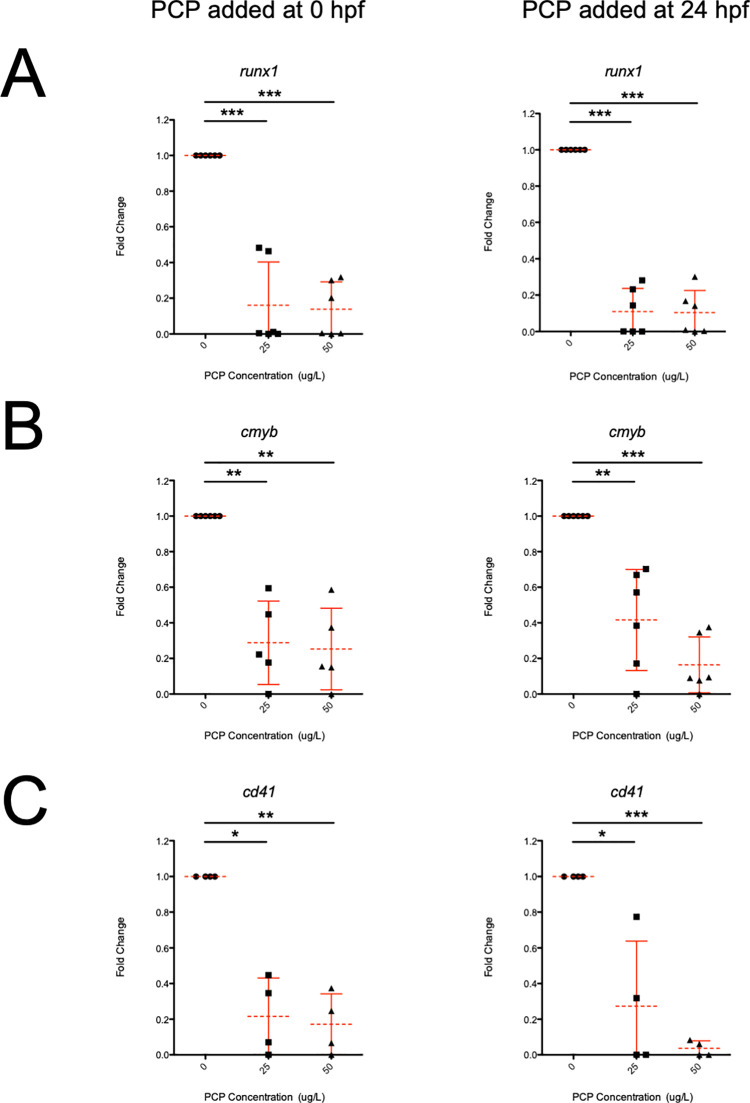
qRT-PCR indicates *runx1*, *cmyb*, and *cd41* expression is lower in PCP treated embryos. Embryos were exposed at 0 (left panel) and 24 (right panel) hpf. Embryos were collected and processed at 48 hpf. qRT-PCR for *runx1* (A), *cmyb* (B), and *cd41* (C) was performed. Each point represents ten embryos randomly selected from live untreated, (circles) 25 μg/L (squares), and 50 μg/L (triangles) PCP conditions. Dashed lines denote mean and error bars represent SD. * denotes p < 0.05, ** denotes p < 0.005, *** denotes p < 0.001.

To analyze the effect that PCP had on upstream stem and progenitor cells in the developing embryo, we administered PCP at 0 and 24 hpf, at concentrations of 0, 25, and 50 μg/L. Using qRT-PCR, we analyzed *runx1*, *cmyb*, and *cd41* which are crucial markers of hematopoietic stem and progenitor cells in zebrafish [21–24]. PCP had a detrimental effect on *runx1*, *cmyb*, *and cd41* expression in live embryos (**Fig 6**), further indicating the severe effect that PCP had on the hematopoietic system.

## Discussion

On November 8, 2018, a faulty transmission line started the Camp Fire in Northern California’s Butte County, which quickly spread to 620.5 km^2^ and destroyed more than 18,000 structures. Eighty-five people were killed and overall costs totaled $16.65 billion. It was the deadliest and most destructive wildfire in California’s history, the sixth-deadliest U.S. wildfire, and ranked number 13 on the list of the world’s most deadly wildfires. Aside from the loss of life, the fire caused severe environmental issues. Smoke from the wildfire traveled over 4800 kilometers, and air pollution was severe in Northern California for weeks. The burning of structures and infrastructure in the area resulted in the release of asbestos, volatile organic compounds, heavy metals, arsenic, dioxins, and other hazardous materials into the air [25]. Importantly, many of these contaminants also likely found their way into drinking water systems and local watersheds.

In this study, we set out to investigate the potential deleterious effects that chemicals released during the Camp Fire could have on the immune system and blood development. PCP, a chlorinated aromatic compound that has been used extensively as a fungicide, was one of the chemicals found in local watersheds after the fire at concentrations in the 10 μg/L range (Zhao et al., in preparation). PCP was first utilized as a wood preservative in the United States, but has since been used in ropes, paints, adhesives, canvas, insulation, and brick walls [26]. In 1984, its use by the general public was restricted, and its application was limited to utility poles, railroad cross ties, fence posts, and wharf pilings [15]. Case studies in the 1980’s and 1990’s indicated that there was likely a link between PCP and hematopoietic cancers and soft-tissue sarcomas [reviewed in 15]. In the 1990’s, PCP was classified as a “possible human carcinogen” by the U.S. Environmental Protection Agency (EPA), based on animal studies. Further studies indicated a strong link between PCP exposure and hematopoietic cancers [15]. There has been no link of PCP to birth defects [27], although animal studies suggested that PCP exposure decreased the survival of rat offspring and caused decreased maternal body weight [27, 28]. While multiple studies have indicated issues with the adult hematopoietic system [15–17], no studies that we are aware of have examined PCP exposure effects on the embryonic development of the hematopoietic system. As the hematopoietic system is responsible for blood formation for the life of an organism, and affected by PCP when examined later in life, we hypothesized that PCP may negatively affect the formation of the blood and immune system. Such effects could be catastrophic for development and may explain the reduced survival of offspring from previous studies, as a functional hematopoietic system is required for the life of an organism.

Standards for PCP exposure have been changing over time. In the 1993 and 2003 editions of the World Health Organization’s *Guidelines for Drinking Water Quality*, the provisional guideline value in drinking water was 9 μg/L. The EPA set the Maximum Contaminant Level (MCL) to 1 μg/L, while California set the Drinking Water Public Health Goal level at 0.4 μg/L in 1997 [28]. Surprisingly, China still relied on the WHO’s recommendation, and in 2003 the allowable level in drinking water was still 9 μg/L. Evidence exists that indicates PCP in the 1970’s was present at 1–10,500 μg/L levels in freshwater locations throughout the U.S. [29], although levels in the 1990’s were much lower. Still, during the 1990’s many locations across the world had water sources with no detectable levels of PCP all the way up to 7.3 μg/L [29]. In China, most locations had higher levels than Western countries, ranging from 0.01 μg/L to 7.36 μg/L. While standards and regulations on PCP have helped reduce the levels of PCP in water sources, PCP can also be present in the air and in sediment. There are numerous reports of 51.5 μg/L (in the U.S.) and 1233 μg/L (in Canada) PCP in the air of urban areas [reviewed in 29]. And levels in sediment are also detectable, ranging from 0.2–16,900 μg/kg [29]. While clearly governmental regulations in most countries have reduced the possibility of humans being exposed to PCP in the air, water, and soil, climate change and the increase in number and severity of wildfires in the WUI is causing more PCP to be released into the atmosphere in smoke, some of which is bound to settle in sediment and water sources. While once only a threat to firefighters, combusted PCP in smoke is now a threat to anyone in the area of massive fires that routinely cover hundreds of thousands of acres throughout the Western U.S. (and other countries) every fire season. In essence, even though the levels of PCP detected in the waterways of Butte County were in the 10 μg/L range, we decided to also look at higher concentrations, with the assumption that the levels that were measured were likely much lower than the amount of PCP actually released into the local environment. Regardless, the ranges of PCP that we examined in this study clearly have physiological relevance to exposures experienced by human beings.

To conduct this study, we developed a workflow of experiments (**S1 Fig**), examining zebrafish embryo survival, morphology, and numbers of erythroid and myeloid cells present. We also examined gene expression for the key hematopoietic transcription factors *runx1* and *cmyb*. Additionally, we examined *cd41*, an integrin expressed on early HPSCs during development. All of these measurements were negatively impacted by the addition of PCP to embryos during their development.

First, we examined the survival of zebrafish embryos. When no PCP was added, all fish survived. When we tested 10 μg/L, we saw no effects on survival. Due to that fact, we increased the levels to 25 μg/L. When 25 μg/L PCP was added, regardless of when it was added during development, the survival rate decreased to between 45–95%. Adding 50 μg/L was even more toxic, with 25–55% surviving, followed by 75 μg/L with 10–30% survival. However, 100 μg/L was the most severe, with only 5–20% embryos surviving. To assess if animals were alive, we used some common measurements; fish had to have a heartbeat and circulating fluid and cells in their vasculature, as well as movement of the pectoral fins and reaction to external stimuli. If fish passed these criteria, they were examined further for hematopoietic defects. In essence, the number of hematopoietic defects in these animals is likely an underestimate, as only healthy animals were observed. The only morphological issue that we observed in fish that were included in the hematopoietic studies were bent tails. While it is impossible to determine if fish were in the process of dying, these steps reduced the chance that these hematopoietic abnormalities were solely due to fish death and developmental delay.

While it is difficult to determine the cause of death for the animals exposed to PCP, previous transcriptomic studies indicate that embryonic fish exposed to PCP experience severe hypoxia and issues with somitogenesis [30]. Other signaling pathways involving metabolism and membrane transport were also altered after PCP exposure [30]. We did observe similar effects in our study, even though animals in those studies were exposed to higher concentrations of PCP. Routinely, we observed issues with body axis curvature, irregular coloration, and necrotic tissue that indicated there were issues with cell adhesion. On most embryos, the tissue was more white and cloudy, and the tissue seemed to fall apart easily, especially with higher concentrations of PCP. Importantly, we also saw small, malformed eyes, as well as issues with melanocyte migration. Overall, these abnormalities indicated that not only mesoderm, the germ layer that gives rise to blood, was affected, but ectoderm was also negatively affected. The epithelial lining around the organism, as well as the formation and migration of neural crest cells (indicated by the melanocyte defects) all derives from ectoderm. Many of these defects were in accordance with prior studies that indicated issues with eye formation and organogenesis after PCP exposure [30]. Gene expression involved in cell adhesion and somitogenesis (which could cause necrosis and axis curvature, respectively) was altered in prior studies, and likely explains some of these morphological defects [30]. Regardless, it is clear that PCP causes unfavorable effects on multiple different tissues derived from multiple germ layers in the developing zebrafish. This is interesting data, as PCP has not been previously tied to birth defects, but at the concentrations examined we saw numerous developmental issues. Further studies on these defects are warranted and should help inform safe levels of PCP in the environment.

After determining that fish could survive PCP treatment, we examined red blood cells in the remaining live animals. Erythrocytes are one of the first blood cell types to arise in the embryo [31, 32], and play an essential role in the function of the animal, as they are essential for transporting oxygen to distant tissues; without red blood cells zebrafish can only survive about one week. Red blood cells arise in two distinct waves. The first wave of hematopoiesis is characterized by red blood cells differentiating directly from mesoderm; this occurs before 30 hpf. After this time point, red blood cells differentiate from hematopoietic stem and progenitor cells [21, 24]; this is how red blood cells are produced for the remainder of the organism’s lifespan. We observed that even at low concentrations of PCP (25 μg/L), red blood cell formation was negatively affected. When added at 0 hpf, almost 50% of fish had decreased blood, and 20% had no blood at all. This negative effect decreased slightly when PCP was administered at 24 hpf, perhaps due to the fact that the first wave of hematopoiesis is more sensitive to the effects of PCP. However, even when administered at 24, 48, and 72 hpf, 50% of embryos had reduced or absent red blood cells, indicating that PCP was having a negative effect on stem and progenitor cells affecting erythroid production. Examining 50 μg/L PCP shows similar results; when administered at 0 hpf, about 65% of embryos had less blood, and about 15% had no blood present. After 24 hpf, the number of embryos with altered blood formation somewhat decreased. The trends with 75 and 100 μg/L indicated that these levels of PCP severely affected blood development. Even though there were some differences in the timing of when PCP affects red blood cell production, it appeared that the first and second waves of red blood cell formation were severely affected in the developing embryo.

Neutrophils are another essential cell type present in the developing embryo, which are primarily responsible for the phagocytosis of bacteria. In zebrafish, they reside in the tissue and can be easily visualized and enumerated. Neutrophils, like red blood cells, also arise in two waves, with similar timing. We observed a decrease of neutrophils in live animals when PCP was added at 25 μg/L at all timepoints. We observed the same with 50 μg/L PCP. At 75 and 100 μg/L PCP, however, we failed to see any animals with myeloid cells when PCP was added at 0 hpf. Later time points showed some animals with discernable myeloid cells, but they were reduced in number. This indicates that the higher concentrations of PCP administered had a more severe effect on the primary wave of myelopoiesis than on the later stages. However, it is clear that all concentrations of PCP administered at all timepoints had negative effects on the number of neutrophils present in the developing zebrafish.

Previous studies indicate that PCP alters gene expression [30, 33] so we examined its role in regulating key hematopoietic-specific transcripts. One of the genes that we examined was the transcriptional regulator *runx1*, present in hematopoietic stem and progenitor cells. The reason for examining *runx1* is due to the fact that during the second wave of hematopoiesis, HSPCs arising from hemogenic endothelium all express *runx1* during their development [34]. In fact, *runx1* expression is required for the epithelial to hematopoietic transition during the formation of stem and progenitor cells in the dorsal aorta [23, 24]. When PCP was added at either 24 or 48 hpf, it had an extremely negative effect on the expression of *runx1*. To confirm that this was not just a reduction in *runx1*, we also examined the hematopoietic transcription factor *cmyb* and the integrin *itgba2* (known as *cd41*). All of these genes are early markers essential for embryonic hematopoiesis [1, 3, 21–24]. As mentioned before, 24–48 hpf is a critical window during development whereby stem and progenitor cells are being generated and expanded [21–24]. Issues in the generation of the proper number of progenitor cells in development would likely have severe consequences long-term in the survival of these embryos.

Overall, we show that PCP had adverse effects on the survival and development of zebrafish. If fish survived treatment with PCP, their development was negatively affected, especially hematopoietic development. We saw a decrease in stem and progenitor cells, which likely caused a decrease in the production of myeloid and erythroid cells. However, adding PCP at later timepoints also reduced myeloid and erythroid cells, so PCP also likely affects their homeostasis. Regardless, there was a severely negative effect on the hematopoietic system. As PCP is getting released into the waterways after destructive urban fires, these studies should be used to evaluate 1) if PCP should continue being used as a wood preservative, 2) if PCP concentrations for wood preservation are simply too high to be safe in the environment and for wildlife and human exposure, and 3) if there are alternative, less-toxic chemicals that can be used as wood preservatives instead of PCP. As the rate and severity of wildfires increases, especially in the Western United States, it behooves us to study these potentially toxic chemicals that can be released during these events and try to mitigate their negative long-term effects on the environment.

## Supporting information

S1 Fig Generalized workflow of experiments. (TIFF)

S1 File Statistical analysis of data. (XLSX)
