# Supplementary figures and images for: Pentachlorophenol has significant adverse effects on hematopoietic and immune system development in zebrafish (Danio rerio)
Source: PLoS One. 2022 Mar 25;17(3):e0265618. doi: 10.1371/journal.pone.0265618 (PMC8956169; doi:10.1371/journal.pone.0265618)

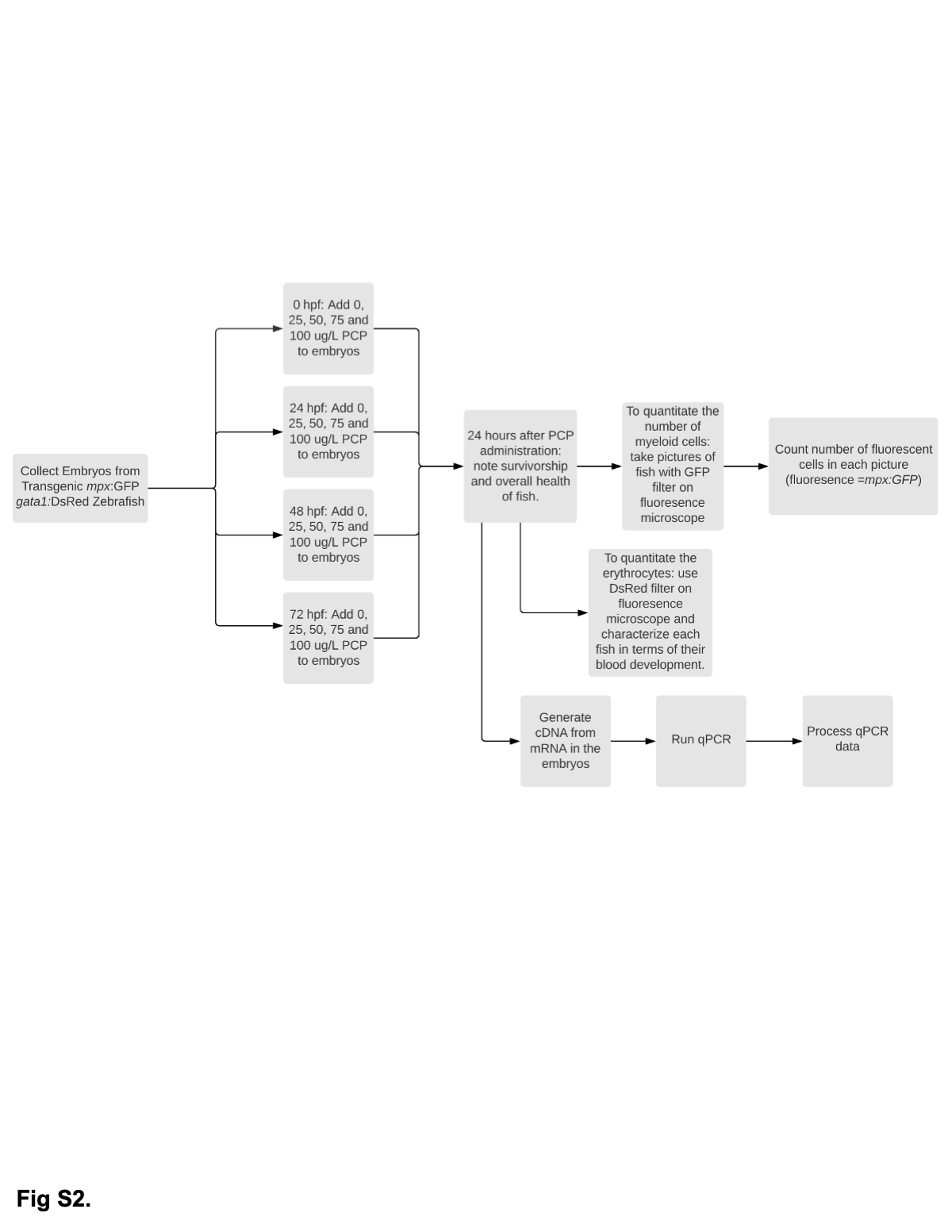

Supplement: S1 Fig — (TIFF) [file pone.0265618.s001.tiff]
